# Baby Friendly Spaces+: a process evaluation of an integrative health approach for lactating women and their babies in Nguenyyiel refugee camp, Gambella, Ethiopia

**DOI:** 10.1080/16549716.2025.2563422

**Published:** 2025-10-27

**Authors:** Molly E. Lasater, Getachew M. Woldeyes, Karine Le Roch, Ohemaa B. Poku, Xuan Phan, Anvita Bhardwaj, Geta Kassa, Andy Solomon-Osborne, Sarah M. Murray

**Affiliations:** a Department of Mental Health, Johns Hopkins Bloomberg School of Public Health, Baltimore, MD, USA; b Mental Health and Care Practices, Gender and Protection, Action Against Hunger, (Action contre la Faim; ACF), Gambella, Ethiopia; c Mental Health and Care Practices, Gender and Protection, Action Against Hunger, (Action contre la Faim; ACF), Paris, France

**Keywords:** Refugee, maternal mental health, Ethiopia, South Sudan, babies

## Abstract

Poor maternal mental health has been shown to impact child health and development. Yet, there is a gap in research on integrated maternal and child care models in humanitarian settings. Action Against Hunger developed the Baby Friendly Spaces (BFS) program which bridges this gap by delivering evidence-based psychosocial support for young children under 2 years and their mothers. This multi-method study was conducted in Nguenyyiel refugee camp (Ethiopia). South Sudanese mothers were assessed at baseline and after 12 weeks on primary outcomes using the *Kessler* Psychological Distress Scale (K6+), PTSD Checklist (PCL-6), and Patient Health Questionnaire (PHQ-9). Secondary outcomes included mother–child relationships, breastfeeding practices and child nutritional status. Qualitative interviews explored whether and how BFS participation is related to maternal and child health outcomes. We included 201 mother and child dyads from the BFS program. Significant improvements were observed for all maternal mental health indicators as well as for mother–child relationships, breastfeeding practices, and child nutritional status. While mothers voiced satisfaction with the BFS program, they also expressed the need for additional material goods. Our study shows that BFS proves to be a successfully integrated program that improves maternal mental health, mother–child relationships, and childcare practices.

## Paper Context

**Main findings:**

Participation in the Baby Friendly Spaces program resulted in statistically significant reductions in all mental health and functioning outcomes, as well as improvement in breastfeeding and most child-care practice, and mothers described positive experiences participating in the Baby Friendly Spaces program and a desire for increased material support.

**Added knowledge:**

This study adds to the growing body of evidence in humanitarian settings highlighting the importance of integrating psychosocial programming into maternal and child health services

**Global health impact for policy and action:**

Future programming must focus on strengthening training, supervision, and capacity of providers, and invest in effective monitoring and evaluation systems to support the implementation and tailored delivery of effective services in order to have optimal health impacts among beneficiaries.

## Background

Exposure to potentially traumatic events and stressors in humanitarian contexts greatly increases the risk of psychosocial distress, associated functional impairment, morbidity, and mortality [1,2]. Among pregnant women and mothers, the psychosocial impacts of traumatic exposures are also demonstrated risk factors for child undernutrition, poor cognitive, motor, and socio-emotional developmental outcomes [3–9]. Moreover, childhood undernutrition and morbidity have been observed to negatively impact developmental trajectories into adulthood, ultimately impacting educational attainment, employment opportunities, and perpetuating a cycle of poverty [1,9,10].

Despite the interrelationship of maternal and child health, there is a prominent gap between research and evidence in the delivery of integrated maternal and child health services in emergency settings [11,12]. One systematic review of psychosocial support (PSS) interventions in humanitarian contexts found very limited evidence for parent-child integrated PSS programs despite their promise as a sustainable approach to improving psychosocial wellbeing of both target groups [13]. This review also noted that while individual-focused PSS were most frequently studied, they posed evaluation challenges as they were designed to be broad in nature (i.e. psychoeducation, mindfulness) and flexible in their use [13]. Moreover, there are critical challenges in delivering care in humanitarian settings including the potential for ongoing conflicts, extreme weather conditions (e.g. drought, floods, extreme heat), human resource and medication shortages, and limited financial resources [14]. These same challenges can also create barriers to carrying out much-needed research on the effectiveness of interventions in humanitarian settings.

In response to the mental health and psychosocial needs of mothers and children in emergency settings, Action Against Hunger (ACF) developed the Baby Friendly Spaces (BFS) program [15]. BFS delivers care practices support for pregnant and lactating (production and secretion of breast milk) women, caregivers, and infants and children under 2 years [5,16] including education related to breastfeeding and feeding, hygiene practices, child play and stimulation, parenting skills, and PSS to enhance mothers’ wellbeing and skills to care for their children [2]. At the BFS center, pregnant and lactating women receive an initial assessment with a trained psychosocial worker (PSW) using standardized instruments to identify specific psychosocial and care practice needs and are then referred to scheduled BFS activities depending on their specific needs. BFS is designed so that women can drop in and attend activities as they wish but they are encouraged to attend at least weekly alongside supplemental food distribution appointments. The program has been implemented in many emergency settings including the Nguenyyiel Refugee camp in Gambella Ethiopia, where a 2019 survey found that nearly half of all respondents (45%) reported experiencing mental health symptoms either sometimes or often that were severe enough to cause impairment in daily functioning [17].

In order to address gaps in knowledge of integrated maternal and child PSS and nutrition interventions in humanitarian contexts, we conducted a multi-method formative process evaluation of the BFS program among South Sudanese refugee mothers with children under age two, residing in the Nguenyyiel refugee camp in Gambella, Ethiopia. We aimed to better understand BFS implementation related to uptake, acceptability, and attendance, and assess whether and how BFS participation is related to improved maternal and child psychosocial outcomes.

## Methods

### Setting

This study was conducted in Nguenyyiel refugee camp, Gambella, Ethiopia, from 6 October 2018 to 6 April 2019. This camp was established in 2016 in response to the South Sudanese civil war. By April 2019, approximately 74,095 South Sudanese refugees were living in Nguenyyiel, 91% of whom were of Nuer ethnicity [18] and over two thirds of whom were under age 18 [19]. ACF was the nominated provider of child and caregiver nutrition-related services in Nguenyyiel, offering nutritional services, malnutrition stabilization centers, BFS, and livelihood activities.

### Study procedures

Data were drawn from three sources: (1) a prospective quantitative survey administered to women at the time of enrollment in BFS and again 12 weeks later; (2) embedded qualitative research with BFS program participants, non-participants, and BFS staff; and (3) routine program monitoring.

### Eligibility and recruitment

To be eligible for study participation, women had to be aged 18 or older, have a child under age two, and be currently breastfeeding. BFS staff members were eligible for participation if they were aged 18 or older and working in the Nguenyyiel refugee camp for at least 1 month. BFS staff members were informed of the study during a staff meeting, and those who were eligible and indicated an interest were referred to a trained data collector who obtained oral informed consent. Women were recruited during their enrollment at the BFS center. Women who met the eligibility criteria were informed of the study, and those who indicated interest in participation were referred to a trained data collector who obtained oral informed consent and administered the baseline survey. Women participating in the qualitative interviews were recruited by trained data collectors using BFS monitoring data and by going door-to-door to identify women who had never participated in BFS. Women who met the eligibility criteria were informed of the study, and those who indicated interest in participation provided oral informed consent. Interviews were either conducted following consent or were scheduled for a later time, depending on participant preference.

#### Quantitative measures

##### Socio-demographics

Women self-reported their age (in years), ethnicity, marital status (dichotomized to currently married or not), years of education completed (dichotomized to any formal education vs. none), number of people living in their home, the number of children for whom they are responsible, work status (dichotomized as paid or self-employed work vs. keeping home or student), the number of years living in the refugee camp, type of roofing (thatch, iron sheets, tent) and frequency of meat consumption (dichotomized to whether or not they eat meat less than once a month). Women indicated if they were currently pregnant, the sex and age of their child under age two, breastfeeding status including frequency per day and duration per session (less than 10 minutes, 10–19 minutes, 20 minutes or more), and past day exclusive breastfeeding of children under 6 months.

##### Functioning

To assess functional impairment, the 12-item Disability Assessment Schedule 2.0 (WHODAS 2.0) [20] was administered asking respondents to indicate how much difficulty they have due to health conditions over the past 2 weeks, ranging from 1 (none) to 5 (extreme or cannot do). Health conditions refer to diseases or illnesses, or other health problems that may be short or long lasting; injuries; mental or emotional problems; and problems with alcohol or drugs.

##### General distress

General distress was measured using the Kessler Screening Scale for Psychological Distress (K-6) [21] which asks six questions about sadness, nervousness, restlessness, hopelessness, feeling everything is an effort, and worthlessness in the last 2 weeks on a five-point Likert scale from 1 (all the time) to 5 (none of the time).

##### Depression

Respondents indicated the number of days over the past 2 weeks they experienced each depression-related symptom on the 9-item Patient Health Questionnaire (PHQ-9) [22] using a 4-point Likert scale from 0 (not at all) to 3 (nearly every day).

##### Post-traumatic stress disorder (PTSD)

PTSD symptoms were measured using the PTSD Checklist-Civilian six-item version (PCL-6) [23]. Respondents were asked to indicate how bothered they were by problems and complaints over the past 2 weeks on a five-point scale from 1 (not at all) to 5 (extremely).

##### Child care practices

Mother-child interactions were assessed using an observation checklist adapted from the UNICEF MICS6 Questionnaire for Children Under 5 [24] based on four minutes of unstructured play between the mother and child.

##### Breastfeeding behaviors

The World Health Organization (WHO) B-R-E-A-S-T- Feed Observation form was used. This is a checklist comprised two scales measuring signs that breastfeeding is going well (6 domains, 26 items) and of possible difficulty (6 domains, 27 items) [25]. Signs that breastfeeding is going well include: body position (5 items), responses (6 items), emotional bonding (3 items), anatomy (4 items), suckling (7 items), and time spent (1 item). Signs of possible difficulty include: body position (5 items), responses (6 items), emotional bonding (4 items), anatomy (4 items), suckling (7 items), and time spent (1 item).

#### Qualitative study

Semi-structured in-depth interviews were conducted with 36 mothers (*n* = 12 who completed the BFS program; *n* = 12 who dropped out; *n* = 12 who never participated) by two trained female Nuer speaking research assistants. Interviews were conducted at BFS centers and explored mothers’ psychosocial needs, experiences with the BFS program, and barriers to participation. In-depth interviews with BFS staff (*n* = 8) explored experiences delivering the BFS program, challenges in program delivery, and solutions for identified challenges.

### Data analysis

#### Quantitative study

We used descriptive statistics to summarize participant characteristics reported at baseline and program monitoring data on women’s engagement in BFS activities. Student’s t-tests and chi-squared tests were used to assess whether demographic or outcomes of interest assessed at baseline were associated with early BFS program dropout (failing to complete more than one session) or program completion (completing five or more sessions). Data from the prospective quantitative study were plotted over time to examine overall patterns of health and wellbeing among women who enrolled in BFS.

We conducted paired Student t-tests and McNemar’s tests to compare scores on mental health scales, breastfeeding practices, and mother–child interactions at baseline and follow-up among all participants. We then conducted separate maximum likelihood estimated random effects linear or logistic regression models with each mental health scale as an outcome with time (baseline or follow-up), number of BFS visits, and a visit number by time interaction term included as predictors, and adjusted for potential confounders (age in years, employed or not, and married or not at baseline). All continuous independent variables were centered at the mean. To account for loss to follow-up, we included an inverse probability weight generated using any baseline demographic or outcome variable associated with loss to-follow up (i.e. the participant did not complete an endline survey) at a significance level of *p* < .10. Variables meeting this criterion included time in camp, baseline PHQ-9 score, baseline functioning score, whether the caregiver smiles or laughs at the child during the evaluated interaction, and number of anatomy-related signs that breastfeeding was going well. The weight was centered and included as a covariate due to challenges with conducting multilevel analyses with inverse probability weighting. We used a Hausman test to assess if a random effect for center was necessary for a model with our primary outcome, depression assessed via the PHQ-9; the test was non-significant (*p* = .06). Thus, we controlled for center as a covariate in all models and used robust variance estimation. To assess moderation by participation in specific BFS activities, we repeated the above models without the interaction term for number of visits and time but with the addition of whether the most commonly implemented activities (focus group discussions, relaxation, and psychological consultation) were received or not and an interaction term between time and participation in the activity, with separate models per activity evaluated.

#### Qualitative study

All interviews were audio recorded and transcribed directly from Nuer into English by research assistants. Analysis of interviews began at the start of data collection. Initial activities included memo writing and debriefing sessions among the research coordinator, research assistants, and first author daily during data collection. Emergent findings and themes were discussed during these sessions and informed subsequent interviews and analyses. Data were inductively coded by MEL and OEP, and analyzed using a thematic analysis approach to identify key themes related to the overall study aims.

### Ethical considerations

This study received ethical approval from the Johns Hopkins Bloomberg School of Public Health Institutional Review Board (#8698) (USA), and the Ethics Committee of Jimma University (#035/10) (Ethiopia). All respondents provided oral informed consent.

## Results

### Sample characteristics at baseline

The study sample consisted of 201 mother–child dyads attending the BFS program (Table 1). At baseline mothers who attended BFS activities were 25.3 (SD = 5.2) years old and had lived in the camp for 1.6 years (SD = 0.7) on average. Most mothers were married (62%), had no formal education (62.2%), and maintained their homes as their primary occupation (89.5%). Average household size was 7 (SD = 2.2) people, and mothers were responsible for an average of 4.7 (SD = 1.8) children. Among children eligible for BFS, mean age was 7.4 months (SD = 6.2), and 58.7% of children were female. On average, children breastfed 9.9 (SD = 3.2) times a day with most breastfeeding sessions lasting under 10 minutes (66.2%). Among children under 6 months, 4% were not exclusively breastfed in the past day. Most women lived in homes with thatch roofing (58.2%), used firewood for fuel (94.5%), and ate meat less than once a month (86%).

**Table 1.** Demographic characteristics at baseline (*n* = 201 mother–child dyads).

*Mother*	
BFS center attended, n (%)	
Center 1	50 (24.9)
Center 2	71 (35.3)
Center 3	56 (27.9)
Center 4	24 (11.9)
Ethnicity, n (%)	
Low	64 (31.8)
Gajak	66 (32.8)
Gajiok	66 (32.8)
Gaguang	5 (2.5)
Age, mean (sd)	25.3 (5.2)
Education, n (%)	
None	125 (62.2)
Some primary	53 (26.4)
Completed primary	9 (4.5)
Some secondary school	14 (7.0)
Marital status, n (%)	
Never married	3 (1.5)
Currently married	123 (61.8)
Separated or divorced	53 (26.6)
Widowed	20 (10.1)
Occupation, n (%)	
Paid work or self employed	17 (4.0)
Student	4 (2.0)
Keeping house	179 (89.5)
Type of roof, n (%)	
Thatch	117 (58.2)
Iron sheets	1 (0.5)
Tent	83 (41.3)
Fuel used, n (%)	
Firewood	190 (94.5)
Charcoal	11 (5.5)
Family eats meat less than once a month, n (%)	172 (86.0)
Number of children responsible for, mean (sd)^a^	4.7 (1.8)
Number of people in household, mean (sd)	7.0 (2.2)
Years lived in camp, mean (sd)^b^	1.6 (0.7)
*Child*	
Sex of child eligible for BFS (e.g. under age 2), n (%)	
Male	83 (41.3)
Female	118 (58.7)
Age of child eligible for BFS in months, mean (sd)^c^	7.4 (6.2)
Child under 6 months not exclusively breastfed in past day^d^, n (%)	4 (4.1)
How long the child breastfeeds per session, n (%)	
Less than 10 minutes	133 (66.2)
10–19 minutes	60 (29.9)
20 minutes or more	8 (4.0)
How often does the child breastfeed per day, mean (sd)	9.9 (3.2)

^a^Missing for one participant; ^b^Missing for 16 participants; ^c^Missing for 2 participants; ^d^Out of 97 children under 6 months old, with four participants missing a value.

### Expressed needs of South Sudanese mothers

Mothers described past traumatic experiences and current daily stressors in their lives, beginning with the destruction of their villages, livelihoods, and deaths of family members, to the violent conditions fleeing South Sudan, and finally new challenges and daily stressors in the camp. Mothers described a very high-stress context in the camp characterized by food, water and medication shortages, tensions among ethnic groups, and the inability to meet their basic needs. When speaking of recurrent food shortages one mother said, ‘we do not have enough food to eat so we do not produce enough milk for the kids to suck.’ All mothers voiced an urgent need for basic material goods such as, soap, clothes, towels, sauce-pans, water collection cans, and mosquito nets. Moreover, mothers described an inability to rest or care for themselves due to the demands of caring for their family, including, maintaining and cleaning the home, collecting firewood and water, caring for their children, preparing meals, bathing young children, and washing clothes. These stressors and responsibilities were described as contributing factors to their experiences of distress:

I just think of all the struggles I went through, now, in my house if my kids are lacking anything I feel depressed and sad because everyone looks up on me to provide them with all their needs.

### BFS engagement

Engagement in BFS activities is summarized in Table 2. The mean number of BFS visits among all participants was 3 (SD = 2). Nearly a quarter (23.4%) of women completed 5 or more sessions, and the maximum number of sessions attended was 9. On average women participated in 1.8 (SD = 1.0) different types of BFS activities and ranged from 0 to 5 activities across all participants. Group discussions (69.2%), play sessions (36.8%) and individual psychological consultations (36.3%) were the most attended activities. Among women who participated in group discussions, average attendance for that activity was 2.2 (SD = 1.4) times, while for women who participated in play sessions, the average number of times completing the activity was 1.4 (SD = 0.7).

**Table 2.** Attendance and participation in BFS program (*n* = 201).

Number of types of activities participated in, mean (sd)	1.8 (1.0)
Total time spent in BFS activities in minutes, mean (sd)	22.9 (36.6)
Received psychological consultation, n (%)	73 (36.3)
Number of visits to BFS, mean (sd)	3.0 (2.0)
Completed 1 visit, n (%)	61 (30.4)
Completed 2 visits, n (%)	44 (21.9)
Completed 3 visits, n (%)	12 (15.9)
Completed 4 visits, n (%)	17 (8.5)
Completed 5 visits, n (%)	17 (8.5)
Completed 6 visits or more, n (%)	30 (14.9)
Ever participated in activity, n (%)	
Play, psychoeducation, or child stimulation sessions	87 (43.1)
Breastfeeding support	32 (15.9)
Relaxation exercises	62 (30.9)
Bathing and massaging	35 (17.4)
Group discussion	139 (69.2)

Nearly a third of women (30.4%) dropped out of the BFS program after attending one session, and drop out significantly varied by BFS center (range: 7% to 52%). BFS drop out was higher among women with lower mean depression (*p* = .01) and functional impairment scores (*p* < .001) as compared to those with higher scores at baseline (Table 3). In contrast, almost a quarter of women (23.4%) completed the BFS program by attending five or more sessions. Program completion also varied significantly by center (range 2–54%) and was higher among women with higher mean depression (*p* < .001) and functional impairment (*p* < .001) scores as compared to women with lower scores at baseline (Table 3). On average women who completed BFS reported fewer individuals living in their household than non-completers (*p* = .04) and their children had a larger Mid-Upper Arm Circumference (MUAC) (*p* = .03).

**Table 3.** Baseline predictors of early dropout from the BFS program (*n* = 201).

	Early dropout^a^			T-test or Chi-2	Program completion^b^			T-test of Chi-2
Characteristic	No (*n* = 140)	Yes (*n* = 61)	*p*-value			No (*n* = 154)	Yes (*n* = 47)	*p*-value
BFS center attended, n (%)								
Center 1	24 (48.0)	26 (52.0)				49 (98.0)	1 (2.0)	
Center 2	66 (93.0)	5 (7.0)				33 (46.5)	38 (53.5)	
Center 3	36 (64.3)	20 (35.7)				52 (92.9)	4 (7.1)	
Center 4	14 (58.3)	10 (41.7)	***p* < 0.001**			20 (83.3)	4 (16.7)	***p* < 0.001**
*Mother*								
Age, mean (sd)	25.6 (5.3)	24.6 (5.2)	*p* = 0.24			25.3 (5.5)	25.1 (4.6)	*p* = 0.81
Any formal education, n (%)	53 (69.7)	23 (30.3)	*p* = 0.98			64 (84.2)	12 (15.8)	*p* = 0.047
Currently married, n (%)	84 (68.3)	39 (31.7)	*p* = 0.42			99 (80.5)	24 (19.5)	*p* = 0.08
Currently working, n (%)	14 (82.4)	3 (17.7)	*p* = 0.25			15 (88.2)	2 (11.8)	*p* = 0.37
Type of roof, n (%)								
Thatch or iron sheets	88 (74.6)	30 (25.4)				85 (72.0)	33 (28.0)	
Tent	52 (62.7)	31 (37.4)	*p* = 0.07			69 (83.1)	14 (16.9)	*p* = 0.07
Fuel used, n (%)								
Firewood	134 (70.5)	56 (29.5)				145 (76.3)	45 (23.7)	
Charcoal	6 (54.6)	5 (45.5)	*p* = 0.26			9 (81.8)	2 (18.2)	*p* = 1.00
Eats meat less than once a month, n (%)	121 (70.4)	51 (29.7)	*p* = 0.47			19 (67.9)	9 (32.1)	*p* = 0.25
Number of children responsible for, mean (sd)	4.7 (1.8)	4.8 (1.9)	*p* = 0.87			4.8 (1.6)	4.6 (2.3)	*p* = 0.49
Number of people in household, mean (sd)	6.9 (2.2)	7.1 (2.2)	*p* = 0.59			7.1 (2.1)	6.3 (2.4)	***p* = 0.04**
Years lived in camp, mean (sd)	1.6 (0.6)	1.5 (0.7)	*p* = 0.27			1.5 (0.7)	1.6 (0.6)	*p* = 0.42
*Child*								
Child eligible for BFS is female, n (%)	81 (68.6)	37 (31.4)	*p* = 0.71			93 (78.8)	25 (21.2)	*p* = 0.38
Age of child eligible for BFS in months, mean (sd)	7.7 (6.2)	7.1 (6.3)	*p* = 0.58			7.5 (6.3)	7.3 (5.8)	*p* = 0.85
Child’s MUAC, mean (sd)	135.4 (19.0)	130.7 (17.2)	*p* = 0.09			133.3 (16.3)	139.0 (13.9)	***p* = 0.03**
*Maternal Mental Health*								
Psychological distress (K-6)	9.7 (5.4)	8.5 (5.0)	*p* = 0.13			9.0 (5.1)	10.6 (5.8)	*p* = 0.06
Depression (PHQ-9)	9.4 (4.2)	7.8 (3.9)	***p* = 0.01**			8.3 (4.0)	10.9 (4.3)	***p* < 0.001**
Posttraumatic Stress Disorders (PCL-6)	12.3 (3.9)	11.9 (4.4)	*p* = 0.58			12.0 (3.9)	12.9 (4.6)	*p* = 0.18
Functional impairment (WHO-DAS)	27.5 (9.1)	22.3 (7.2)	***p* < 0.001**			23.9 (8.2)	32.7 (7.6)	***p* < 0.001**

^a^Defined as only attending one session. ^b^Defined as attending 5 or more sessions

Bold indicates that the *p*-value is significant at a level of < 0.05.

### Changes in maternal mental health, functioning, and breastfeeding practices during the program

Significant reductions were observed in all forms of mental distress at follow-up relative to baseline (Table 4). The mean K6 and PCL-6 scores were reduced both by 19% (*p* < .001), while the PHQ-9 score reduced by 23% (*p* < .001) at follow-up. A 15% (*p* < .001) reduction was observed in the WHODAS indicating less functional impairment. Increases were observed in the signs that breastfeeding was going well across all domains at follow-up: body positioning (19%; *p* = .002), response (20%; *p* < .001), emotional bonding (25%; *p* < .001), anatomy (16%; *p* < .001), and suckling (18%; *p* < .001). Significant reductions were also observed in the signs of breastfeeding difficulty at follow-up for all domains: body positioning (27%; *p* < .001), response (35%; *p* < .001), emotional bonding (21%; *p* = .004), anatomy (27%; *p* < .001), and suckling (30%; *p* < .001).

**Table 4.** Change in maternal mental health, functioning and breastfeeding over the course of the BFS program.

Outcome	Baseline (*n* = 201)	Follow Up (*n* = 170)	Mean difference (se)	Percent change	p-value	Possible range
*Breastfeeding*						
Number of signs that breastfeeding is going well						
Body positioning, mean (sd)	2.2 (1.2)	2.6 (1.2)	0.41 (0.13)	19%	p = 0.002	0–4
Response, mean (sd)	3.1 (1.2)	3.7 (1.2)	0.63 (0.15)	20%	p < 0.001	0–5
Emotional Bonding, mean (sd)	1.5 (1.0)	1.9 (0.9)	0.38 (0.10)	25%	p < 0.001	0–3
Anatomy, mean (sd)	2.5 (1.0)	2.9 (1.1)	0.41 (0.11)	16%	p < 0.001	0–4
Suckling, mean (sd)	4.2 (1.7)	5.1 (1.6)	0.93 (0.18)	18%	p < 0.001	0–7
Number of signs that breastfeeding is not going well (difficulties)						
Body positioning, mean (sd)	1.8 (1.2)	1.3 (1.2)	−0.49 (0.12)	−27%	p < 0.001	0–4
Response, mean (sd)	1.8 (1.4)	1.2 (1.1)	−0.64 (0.15)	−35%	p < 0.001	0–5
Emotional Bonding, mean (sd)	1.4 (1.0)	1.2 (0.9)	−0.30 (0.10)	−21%	p = 0.004	0–3
Anatomy, mean (sd)	1.4 (1.0)	1.1 (1.1)	−0.38 (0.11)	−27%	p < 0.001	0–4
Suckling, mean (sd)	2.7 (1.6)	1.8 (1.6)	−0.82	30%	p < 0.001	0–7
*Mother Mental Health*						
Psychological distress (K-6)	9.4 (5.3)	7.7 (4.5)	−1.8 (0.4)	19%	< 0.001	0–30
Depression (PHQ-9)	8.9 (4.2)	7.1 (3.6)	−2.1 (0.3)	23%	< 0.001	0–18
Posttraumatic Stress Disorder (PCL-6)	12.2 (4.0)	10.0 (3.0)	−2.3 (0.3)	19%	< 0.001	6–30
Functional impairment (WHO-DAS)	26.0 (8.9)	22.9 (6.4)	−4.0 (0.6)	15%	< 0.001	12–60

Significant increases were observed in some positive mother–child interactions during the BFS program (Table 5). For positive care practices, at follow-up mothers had an increased odds of talking to her child (OR: 1.9; 95% CI: 1.1, 3.3); interacting with the child to promote development and learning (OR: 2.5; 95% CI: 1.5, 4.2); and, smiling, laughing, caressing, kissing or hugging her child (OR: 1.9; 95% CI: 1.1, 3.2). Increases were noted in the mother looking at her child often (OR: 2.1; 95% CI: 1.0, 4.8) and reporting someone in the household over age 15 who told a story, sang, or played with the child in the past day (OR: 1.2; 95% CI: 0.6, 5.2), but these were not statistically significant. Mothers also reported increased odds of leaving her child alone or with a child under 12 years more than once in the past 2 weeks (OR: 2.8; 95% CI: 1.6, 5.2) at follow-up, but no significant changes were observed in the use of harsh discipline techniques by the mother (OR: 1.0; 95% CI: 0.5, 2.2).

**Table 5.** Change in care practices over the course of the BFS program.

		Follow Up	Odds
	Baseline (*n* = 201)	(*n* = 170)	ratio (95% CI)
Keeps child in visual range and looks at child often, n (%)	144 (85%)	155 (92%)	2.1 (1.0, 4.8)
Caregiver talks to the child, n (%)	110 (65%)	130 (77%)	1.9 (1.1, 3.3)
Caregiver interacts with child to promote development and learning, n (%)	83 (49%)	119 (70%)	2.5 (1.5, 4.2)
Caregiver smiles at the child, laughs with the child, caresses, kisses or hugs the child, n (%)	94 (56%)	118 (70%)	1.9 (1.1, 3.2)
Caregiver spanked or hit the child, or shouted or yelled at him/her, n (%)	17 (10%)	17 (10%)	1.0 (0.5, 2.2)
Yesterday or today, someone in household over 15 told story, sang or played with child, n (%)	108 (64%)	112 (67%)	1.2 (0.6, 2.2)
Left child alone or with child under 12 more than once a week in past 2 weeks, n (%)	51 (31%)	85 (52%)	2.8 (1.6, 5.2)

In adjusted random effects longitudinal linear regression models (Table 6), participants who completed the average number of sessions (3.0) experienced statistically significant reductions in psychological distress (β = −1.61; 95% CI: −2.53, −0.69), depression (β = −1.89; 95% CI: −2.57, −1.20), and post-traumatic stress (β = −2.07; 95% CI: −2.71, −1.43), with the largest reduction found in functional impairment (β = −3.94; 95% CI: −5.14, −2.75). The interaction term between number of visits and time was non-significant in all models, indicating no statistically significant differences in reduction of mental distress or functional impairment comparing individuals who completed additional BFS sessions compared to those who completed one fewer. When examining moderation by participation in specific activities, individuals who participated in relaxation activities experienced less of a reduction in depression compared to those who did not participate in relaxation activities (β = 1.78; 95% CI: 0.28, 3.27), and mothers who received a psychological consultation, compared to those who did not, reported less of a reduction in functional impairment (β = 2.42, 95% CI: 0.10, 4.73).

**Table 6.** Results of random effects longitudinal linear regression models assessing engagement in BFS sessions and activities and change in mental health outcomes from pre- to post-intervention.^a^

	Model 1			Model 2	Model 3	Model 4
*Outcome*	Time	Number of visits	Interaction of time and number of visits	Interaction of time and focus group discussion	Interaction of time and relaxation activity	Interaction of time and psychological consultation
	Beta (se), p-value	Beta (se), p-value	Beta (se), p-value	Beta (se), p-value	Beta (se), p-value	Beta (se), p-value
Psychological distress (K-6)	**–1.61 (0.46), *p* = 0.001**	0.21 (0.24), *p* = 0.37	−0.15 (0.26), *p* = 0.56	−1.40 (1.07), *p* = 0.19	1.09 (1.04), *p* = 0.30	−1.26 (0.99), *p* = 0.20
Depression (PHQ-9)	**–1.89 (0.35), *p* < 0.001**	0.13 (0.17), *p* = 0.43	0.05 (0.22), *p* = 0.82	0.36 (0.76), *p* = 0.64	**1.78 (0.76), *p* = 0.02**	0.12 (0.72), *p* = 0.87
Posttraumatic Stress Disorder (PCL-6)	**–2.07 (0.33), *p* < 0.001**	0.09 (0.17), *p* = 0.58	−0.24 (0.18), *p* = 0.19	−0.50 (0.69), *p* = 0.47	0.48 (0.66), *p* = 0.46	1.34 (0.70), *p* = 0.06
Functional impairment (WHODAS)	**–3.94 (0.61), *p* < 0.001**	0.52 (0.26), *p* = 0.05	−0.42 (0.33), *p* = 0.20	1.02 (1.2), *p* = 0.40	−0.96 (1.23), *p* = 0.44	**2.42 (1.2), *p* = 0.04**

^a^All models included time and number of visits to BFS as a predictor and controlled for age, working (yes/no), married (yes/no) and center. Model 1 includes a time × number of visit interaction; Model 2 a time by focus group participation interaction; Model 3 a time by relaxation activity participation interaction and Model 4 a time by receipt of psychological counseling interaction. Se = robust variance estimated standard error.

Bold indicates that the *p*-value is significant at a level of < 0.05.

### South Sudanese mothers’ experiences with the BFS program

In order to cope with their problems some mothers described seeking support from the BFS program. Mothers spoke very positively of the BFS program and staff. Most mothers said they would recommend BFS,

I will tell her that she would be taught how to protect our children, that she can get soap and baby clothes inside. We get advice even if you have something that makes you emotional, they use counseling, I should tell that is where women drink tea and dance, and they also share their experiences.

Mothers reported attending individual counseling sessions with PSWs and learning about managing their thoughts and emotions; group discussions where they learned about hygiene and sanitation practices for themselves, their children and their home; and breastfeeding support. Both mothers who completed the BFS program and dropped out of BFS described positive changes in their mental health and being able to ‘*forget about their worries*.’ One mother stated,

Yes, there is a difference, because how I came is not how I am now. I have learnt a lot from this program about many things that I didn’t know about before. And I am very much thankful to the organization because I had a lot of stress when I first came but I feel much better and doing very well now because of the counseling I received.

When asked about suggestions for improving BFS, almost all mothers who had either completed or dropped out of BFS reported that they were satisfied with the program. However, several mothers voiced desires for BFS to provide material goods, such as clothing, blankets, and cooking materials, as well as reading and writing education for mothers. Among the mothers who stopped attending BFS, almost all reported that they had lost their ACF identification card and that they would resume attending if they received a new card. Mothers who had never participated in BFS shared that they were new to the camp and did not know of BFS or that they were too busy as reasons for not participating. While most women perceived BFS services to be helpful, they also voiced that they would be more willing to attend if BFS also provided the needed material goods described above.

### BFS staff members needs delivering BFS

BFS staff members described positive experiences and feelings of fulfillment delivering the program. Most staff members described their work as important and meaningful, citing the positive impacts that the BFS program can have on the lives of mothers. However, staff expressed several needs and challenges delivering BFS. The primary need voiced by BFS staff was for regular training and capacity building on intervention implementation including, counseling skills, psychological first aid, problem identification tools, computer skills, reporting and monitoring, and administering the admissions questionnaire. While PSWs shared that they had received a training on providing PSS, many voiced the need for additional and related trainings to strengthen their skills:

The other challenge is as a psychosocial worker, there are areas of improvement that we need to be trained with so that we can be more confident in our work … . Training on stress management activities and counseling skills would help me as well. (PSW)

BFS staff also discussed challenges related to the need for more resources including, more space for activities, computers for reporting monitoring data, and more Nuer speaking staff. Staffing shortages were described as occurring frequently and were particularly exacerbated during periods of ethnic conflict in the camp when Nuer staff were not able to work due to security concerns. Relatedly, language barriers were described as a source of frustration and as a factor challenging BFS service delivery, as Nuer staff were frequently interrupted while delivering BFS services to act as translators. BFS staff explained that these needs and challenges inhibited their ability to meet both the individual and collective needs of mothers and their children attending BFS.

### BFS improvements

BFS staff offered several suggestions for improving BFS services and delivery. Modifications to the BFS admission questionnaire were recommended: it was described as too long, repetitive, and not easily understood by or culturally adapted for use among Nuer women with low literacy levels. One PSW stated,

The admission form is very vast which exhausts the mother during questioning, and she may not quickly understand the questions, hence requiring a lot of time for explanation.

Moreover, staff recommended modifications to the monitoring system so that *‘only relevant information needs to be registered in the registration book’*. A few staff members described the frequent occurrence of reporting errors given a lack of training on reporting program activities and the vastness of information collected as a part of program monitoring:

While reviewing reports from staffs, sometimes the numbers in the report seem unrealistic or exaggerated, the number of participants doesn’t correspond with the total number of sessions conducted, there is a much greater number of participants compared with the expected number in a session. Therefore, this is time taking to go back and cross check and arrange the report accordingly. (BFS supervisor)

Relatedly, staff suggested the use of fidelity checklists to ensure activities are being implemented as intended.

I sometimes see that activities were not being implemented properly … I believe that we can improve the capacity of psychosocial workers’ reporting skills through formal trainings. Secondly, there should be standard checklists where we could use during field observation as monitoring system. (BFS supervisor)

BFS staff also shared several recommendations to improve the engagement of BFS beneficiaries. Given refugee mother’s urgent needs for basic material goods, BFS staff recommended providing mothers with basic goods such as blankets, towels, food, cooking supplies, and soap as an incentive to encourage BFS engagement. One staff member shared:

Our work focuses on bringing behavioral change to beneficiaries, however, because of the refugee’s urgent needs, they always have material expectations regardless of our activities … It makes it difficult for them to participate in all the activities when their expectation is not fulfilled.

Several staff members also described a need to include gender-based violence (GBV) services – in addition to referrals to other NGOs –, a key issue facing many mothers. Staff recommended broadening the scope of BFS services to engage the whole family and target orphaned children who were currently not supported by BFS services. Specifically, staff discussed the need for father-to-father support groups to help fathers cope with stressors, and to raise fathers’ awareness of BFS activities as a means for them to encourage their wives to attend.

## Discussion

We conducted a multi-method process evaluation of the BFS program among South Sudanese refugee mothers with children under age two in Ethiopia to enhance program implementation and assess whether and how BFS participation is related to improved maternal and child health outcomes. Participation in the BFS program resulted in statistically significant reductions in all mental distress and functioning outcomes, improvement in breastfeeding practices, and most child-care practices. Mothers participating in BFS described enjoying the program and BFS staff reported their work to be meaningful and impactful on the lives of mothers participating in the program.

Despite greater BFS engagement among mothers with higher mean depression and functional impairment scores at baseline, there was no statistically significant difference in mental health or functional impairment outcomes associated with number of sessions attended. This points to the importance of controlled evaluations that allow for the assessment of different combinations of components to better understand what works for whom, which can ultimately help to decipher what the active ingredients are of successful psychosocial programs that are multicomponent [26]. This in turn could help make psychosocial interventions like BFS less time and resource intensive and better able to meet the needs of beneficiaries in adaptive ways [26,27]. Moreover, given the observed improvements in breastfeeding and child-care practices in this study, future research should be designed to allow for mediation analyses to assess whether improvements in mental health and functioning lead to improvements in breastfeeding and child-care practices, or vice-versa.

Many interventions, including BFS, are comprised of multiple components or activities (i.e. play sessions, relaxation exercises, group discussions) and are commonly evaluated as a whole intervention package [13,27,28]. Errors in reporting are common due to mismatch in services offered with mothers’ needs. Minimal and effective monitoring systems, that align with an intervention’s program manual and are supported by strong and frequent trainings and supportive supervision are needed to help bridge this mismatch, strengthen key intervention activities, and identify challenges or issues related to intervention implementation [29]. It also points to the need for implementation research that focuses on the training, competency and decision making of providers of psychosocial programs that are often designed to be flexible to meet the needs of a variety of contexts and heterogeneous target populations. For example, a recent pragmatic cluster-randomized controlled trial of BFS among Rohingya refugee mothers in Cox’s Bazaar, Bangladesh, compared BFS treatment as usual (BFS-TAU) with implementation enhanced BFS (BFS-IE) and found significant within-group improvements in BFS-IE for distress, functional impairment, and subjective well-being [30]. Improvements in BFS-TAU were smaller and not statistically significant. The study attributed differences in outcomes to the increased program training and implementation and supervision support among BFS-IE [30]. Tools such as the Enhancing Assessment of Common Therapeutic Factors (ENACT), can be used to assess provider competency, evaluate trainings, and ultimately optimize supervision strategies that support trainee development and fidelity and quality of services delivered when strict manualization is not possible and flexibility is needed to be responsive to a broader set of concerns for the purpose of prevention of distress and promotion of mental health [31]. The uptake of such training and monitoring tools and systems will allow for the examination of implementation factors and clarity regarding the extent to which intervention impacts may be attributed to intervention components themselves, non-specific provider characteristics, or implementation factors related to the intervention, and ultimately strengthening the effectiveness of services more holistically in real-world settings [27,28].

Over the last 30 years, humanitarian organizations have strived for consistent recognition and response to the needs of women in these settings and their sustained access and engagement in basic social and health services [32]. While evidence is mixed regarding improved access, a focus on the needs of women and their family and the implementation of minimum service packages have made services for women more available [32]. However, in our study, 30% of women who accessed BFS stopped attending the program after one session. As was found for participants in this study, women are often responsible for providing for their families during crises and take the lead on finding food, water, shelter, and caring for others [33], and women reported significant unmet needs in these domains. While basic needs such as food, water, and shelter are prioritized within humanitarian settings, a comprehensive assessment of needs that are essential to all aspects of daily living are necessary [34]. While a systematic review examining the impact of food aid on mental health in households with children in high-income settings identified very limited evidence of a relationship between food distribution and mental health [35], in humanitarian settings, the extant challenges faced by women in these contexts regarding food, water, and housing insecurity only exacerbate other challenges faced by women and are known to contribute to poor mental health, and may interfere with their ability to access services consistently [36]. Competing interests and constant change in priorities for women requires humanitarian services to respond to, not complicate the accessibility of resources [33].

In many communities, mothers are the primary caregivers and hence many interventions, including BFS, target mother-child dyads [37]. Yet, there is a large body of evidence that shows that fathers, grandmothers, and other family members play a role in maternal and child health outcomes [37–40]. BFS staff highlighted the need for family focused interventions to help all family members understand care practices and ultimately better support mothers and children. A recent systematic review identified 63 studies examining family-level social and behavioral interventions to improve maternal nutrition or infant and young child feeding practices [37]. The majority of those interventions involved fathers and grandmothers, but many were not designed to engage them specifically [37]. Even then, interventions that engage family members have been shown to have positive impacts in addressing gender norms, women’s empowerment, family communication, and relationship dynamics that help with reducing GBV [41,42].

The findings of this study have important implications for future programming targeting pregnant and lactating women, caregivers, and children. Findings of this study support growing evidence on the interrelationships between maternal mental health and child health. Future programming must embrace this interrelationship and develop programs with a focus on the integrated and joint management of maternal and child health care to improve health outcomes [43]. Moreover, in order to ensure that essential mental health services are delivered to those in need, future programming must be attentive to the importance of measuring maternal mental health [44]. Assessing the burden of mental health problems facing a population can also help guide the allocation of programming resources including, time, financial, treatment, and human resources [44,45]. Lastly, our findings, as well as the findings of the BFS cluster-randomized controlled trial in Bangladesh, reinforce the critical need for future programming to prioritize refresher trainings and ongoing supportive supervision to ensure high-quality implementation [14,30,46]. Tailor made, culturally adapted, and flexible programs such as BFS are not always able to adhere to strict manualization, which may present fidelity challenges. Therefore, future programming requires ongoing training and supervision to support program fidelity, strengthened therapeutic competencies and ultimately, improved health outcomes [47].

### Limitations

This study was conducted in a setting of high adversity, including periods of instability and ethnic conflict in the camp that not only resulted in the suspension of humanitarian operations in the camp at times, but may have restricted women’s movement in the camp, including BFS participation, before and following acute instability [14]. Another limitation of this study is that the quantitative measures included in the questionnaire have not been validated for use in the Nuer language. However, we undertook extensive translation and back translation process of the questionnaire, including having Nuer staff members provide feedback and input. Additionally, repeated exposure to trauma and stress in the camp may have confounded the relationship between BFS participation and the outcomes of interest (mental health and functioning, breastfeeding quality, and child-care practices). This study also lacked a control group, making it difficult to assess the extent to which contextual factors may have mediated changes in outcomes. Lastly, assessing the transferability of our study findings is complex, as the BFS program is meant to be flexible in its cultural and contextual adaptation. Notably, the BFS program has been successfully implemented in diverse humanitarian settings, with careful attention to cultural adaptation, which supports the transferability of our findings to other humanitarian settings. The refugee camp in Gambella shares many key characteristics with many camps that have experienced large influxes of newly arriving refugees. However, the highly adverse context of this setting, characterized by periods of instability and ethnic conflict within the camp, may limit the transferability of the findings to more stable, non-humanitarian contexts. Moreover, given that one component of the eligibility criteria for study participation among women required them to be currently breastfeeding, our findings are likely most transferable to currently breastfeeding women, rather than those who have stopped for any reason, in similar humanitarian settings.

## Conclusion

Findings of this BFS program process evaluation revealed enjoyment participating in and delivering the BFS program, and statistically significant reductions reported by participants in all mental distress and functioning outcomes and improvements in breastfeeding and most child-care practices. Future research efforts examining PSS interventions should incorporate comparison conditions to better understand which intervention components work best, and for whom. Additionally, enhanced training and supervision structures as well as an effective monitoring and evaluation system are needed to support implementation efforts and the tailored delivery of effective services. Continued investment in mixed- method evaluations of PSS interventions are needed to provide the best support and responses to women and young children in humanitarian contexts, and to ultimately improve policy and practice.

## Data Availability

The data that support the findings of this study are available from the corresponding author, KLR, upon reasonable request.
